# Twelve quick tips for deploying a Beacon

**DOI:** 10.1371/journal.pcbi.1011817

**Published:** 2024-03-01

**Authors:** Lauren A. Fromont, Mauricio Moldes, Michael Baudis, Anthony J. Brookes, Arcadi Navarro, Jordi Rambla

**Affiliations:** 1 Centre for Genomic Regulation (CRG), The Barcelona Institute of Science and Technology, Barcelona, Spain; 2 University of Zurich, Zurich, Switzerland; 3 Swiss Institute of Bioinformatics, Zurich, Switzerland; 4 Department of Genetics & Genome Biology, University of Leicester, Leicester, United Kingdom; 5 Department of Medicine and Life Sciences, Universitat Pompeu Fabra, PRBB, Barcelona, Spain; 6 Institució Catalana de Recerca i Estudis Avançats (ICREA) and Universitat Pompeu Fabra, Barcelona, Spain; 7 BarcelonaBeta Brain Research Center, Pasqual Maragall Foundation, Barcelona, Spain; 8 IBE, Institute of Evolutionary Biology (UPF-CSIC), Department of Medicine and Life; SIB Swiss Institute of Bioinformatics, SWITZERLAND

## Introduction

In the age of data-driven biomedical research and clinical practice, the sharing of genomic and clinical data for health research and personalized medicine has become an important contributor to improved diagnosis and treatment. From the data owner’s perspective, potential benefits include improved treatments, personalization of healthcare practice, and more effective control of disease proliferation. However, the requirement for high levels of data security to protect sensitive information presents a barrier to data discovery and sharing [1].

*Beacon* is designed to enable the benefits of data discovery while minimizing the associated risks. It is a Global Alliance for Genomics and Health (GA4GH) **API specification** (see Box 1 for a definition of key Beacon terminology), allowing easy discovery of sensitive data that require controlled (authorized) access [2]. It uses simple concepts and can be adapted to different use cases. The protocol is designed to respond to queries, such as the following:

**Box 1. Key terminology and definitions**

Beacon API specification: An Application Programming Interface (API) protocol that defines a framework for queries containing genomic, phenotypic, clinical, and technical parameters. Documentation: http://docs.genomebeacons.org/

Beacon Reference Implementation: To facilitate the adoption of the Beacon API, the ELIXIR organization funded the development of the Beacon Reference Implementation. This is a specification-compliant free open-source Linux-based Beacon implementation, completed with a set of tools to light a Beacon directly: https://b2ri-documentation.readthedocs.io/en/latest/

Beacon instance: A Beacon instance (or *beacon*, without capitalization) refers to a beacon that is compliant to the Beacon API specification. It can be developed from the specification directly or deployed using the Beacon Reference Implementation.

Beacon deployment: The action of lighting a beacon using the Reference Implementation, as opposed to *developing* a beacon using the Specification.

Beacon network: An aggregation of individual beacons. From a user perspective, it can be queried similarly to a beacon, and the response may report which database each discovered dataset is from. Repository for a Beacon network reference implementation: https://github.com/elixir-europe/beacon-network-backend

“Can you provide data about males, diagnosed with Type 2 diabetes, whose age of onset is below 30 years, and who carry mutations in the APOE gene?”

Depending on the data controller’s preferences over response granularity, the response options range from, “Yes, our data includes one or more” (boolean response), “Yes, we have 125” (count response), to “Yes, and here are some details about the 125 individuals that match your request” (detailed “record level” response).

Discovery is the necessary first step in the sharing and reuse of data and other assets, and Beacon facilitates this by enabling federated discovery in any number of networks, as a complement to unwieldy central catalogs. Many beacons have already been successfully “lit” (deployed) across the globe (https://public.tableau.com/app/profile/elixir/viz/ELIXIRBeaconNetwork/Sheet1). This article is written to support data owners that might be interested in **deploying a beacon** to make their data discoverable while keeping them secure. Whatever your background is, this article will provide you with some tips to get you started. Specifically, we will review important steps to complete—and pitfalls to avoid—when deploying a beacon.

### Tip #1: Evaluate the value of your data to your research or clinical domain

Deploying a **Beacon instance** will help you keep the data both as open as possible and as restricted as necessary (Tip #9). That said, you might want to take into account the *perceived* value of your dataset, which depends on the target communities. For example, a dataset on the Bordurian population could be of interest for Bordurian researchers, but a dataset focusing on Alzheimer’s disease would interest a different and wider community.

Ask yourself: What special value does your dataset bring to one or more given communities?

Identify the variables that are the most relevant for users who would be interested in these data (age of onset, ancestry background, treatments taken, body measures, etc.), as these aspects will be the most useful for your beacon to receive queries about.

### Tip #2: Understand the Beacon concepts and choose your Beacon options

The power of Beacon stems from its flexibility: It uses simple concepts and can be adapted to different use cases. This means you will need to understand the available options and think about the optimal combination needed for your specific use case. Among the parameters to consider are dataset sensitivity, applicable regulations, conditions in the agreement with the data donors, available human and infrastructure resources to deploy the beacon instance, etc.

Get started: To get a better understanding of GA4GH Beacon, consult https://docs.genomebeacons.org/.

### Tip #3: Get stakeholders support

In the case of lighting a beacon for potentially sensitive data, it is necessary that data controllers (backed by ethics committees, data protection officers, etc.) authorize and actively support the initiative. System administrators and network security officers must also participate, as well as people or institutions that will supply the required funding. If you don’t obtain all this required support for deploying a sensitive data beacon, the project will probably fail.

Take action: Explain to the stakeholders the value of showcasing the dataset (Tip #1) and the principles of Beacon (Tip #2).

### Tip #4: Assess your human and infrastructure resources

Deploying a beacon is not technically demanding (Tip #6), but populating it with an appropriate version of your data will require some data transformation and management. Furthermore, configuring the beacon for your use case will require some planning and understanding of the Beacon concepts (Tip #2) and implementation (Tip #6). Both technical and infrastructure resources should be assigned to the Beacon deployment, and some team members must be responsible for learning about Beacon, as well as deploying and configuring your Beacon instance.

Plan your beacon deployment. Deploying a Beacon is a project in itself: Include it in your regular planning and make sure you have the resources needed for every step of the deployment process. The present tips can be used to create the initial timeline.

### Tip #5: Start humble

Beacon is flexible (Tip #2), allowing the Beacon instance to incorporate and make discoverable all or only some of the Beacon entity Model types (i.e., individuals, biosamples, genomic variations, runs–wet lab–, analysis–dry lab–, datasets, and cohorts). You could, for example, start with a beacon that describes only the statistical attributes of your cohort (for example, age pyramid, conditions, geographical regions…) or a beacon that includes only biological sample attributes. Your beacon may also be configured to return only “yes/no” or counts of results to queries.

Start small, then grow. Before moving to a more comprehensive edition of your beacon, start sharing the information that would attract more users while requiring less effort. Focusing on the variables identified in Tip #1 as terms for query, and returning count responses (“how many?”) is a popular starting point.

### Tip #6: Deploy an out-of-the-box beacon

Several implementations of the GA4GH Beacon specification have now been implemented, with the ELIXIR **Beacon Reference Implementation** (RI) being one of them [3]. While the RI is not designed for lighting heavy-load production beacons, it is very useful for deploying a prototype, and, thereby, to initiate the process of working toward a robust production Beacon instance.

Deploy a beacon prototype: https://b2ri-documentation.readthedocs.io/en/latest/.

### Tip #7: Decide which query terms are within scope

Beacon instances only respond to queries about terms they understand. A beacon on cancer data would answer “no” to any query about psychiatric disorders or autoimmune diseases. Before doing a query, a beacon user (for example, a researcher querying the beacon) or client (for example, a beacon aggregator or other software; see Tip #12) needs to know the valid concepts within the intended scope of the Beacon. Therefore, beacons are required to provide a list of the terms available for filtering (*filtering terms*). These terms would be the concepts identified in Tip #1 that can be filtered for. Although custom vocabularies are accepted, the Beacon specification strongly recommends using ontology terms and labels as filtering terms. For example, instead of using “melanoma,” you may opt for NCIT:C3224—Melanoma (or its equivalent in another ontology like SNOMED). To make the list of filtering terms easier to browse, a user interface is usually deployed in front of the beacon server.

Populate the filtering terms list with the attributes and values that should be used to query your beacon, preferably referring to existing ontologies.

### Tip #8: Provide handovers

Beacon provides a standardized way by which datasets can be made remotely discoverable based on their characteristics, by researchers who are looking for such items. Once the data have been discovered or found, the user’s next question is, “how do I access the actual data?” The Beacon specification includes a feature named *handovers* to facilitate the actual transfer of data or additional information. Handovers allows the beacon server to attach relevant information (usually in the form of URLs) to a response. For example, “Here is the data access request form,” “More details on this mutation can be found here,” or "Here is the VCF file containing the genomic variants matched by the query.”

Evaluate which “next steps after discovery” make sense for your users and which handovers could be possible or necessary for these next steps. Include them in your beacon backend code.

### Tip #9: Plan the security aspects

The Beacon specification recommends secure settings by default, which deployers do not necessarily need to follow. A beacon service may require a user to log in (authentication) and allow its users to only query specific datasets that have been preauthorized for a particular user or user group. While the choice of an authentication and authorization infrastructure is out of scope for the protocol, we recommend to follow standard security implementations. For example, GA4GH and ELIXIR recommend OpenID Connect (OIDC; https://openid.net/) on top of OAuth 2.0. The Beacon RI includes Keycloak (https://www.keycloak.org/), an implementation of that protocol. ELIXIR also provides the Life Science Authentication and Authorization Infrastructure (LS AAI; https://lifescience-ri.eu/home.html) as a service.

Furthermore, deploying the beacon in a secure network configuration, where sensitive data is in a secure area, while the service façade is in a public facing area, needs to be configured responsibly by the network stewards.

Choose and document the security aspects of your beacon service carefully; be ready to collect and answer questions about it.

### Tip #10: Check that your informational endpoints are useful

The Beacon specification is designed to support beacon networks (Tip #12). Therefore, a beacon service is required to share some metadata about the service itself, including available entry types and filtering terms, required authentication levels, etc. These metadata are provided through the *informational endpoints*, but these endpoints are simply returning the information configured by the beacon administrator.

Ensure good metadata. Review the informational endpoints and check that the data included in them are correct and complete.

### Tip #11: Verify your beacon

You can verify whether your beacon matches the Beacon specification using the Beacon Verifier. A greenlight from the Verifier provides a guarantee that your beacon implementation complies with the Model and Framework.

Verify your beacon compliance using this link: https://github.com/EGA-archive/beacon-verifier.

### Tip #12: Join a beacon network

Each distinct Beacon provides a showcase that will become more and more popular as it joins wider infrastructures named *beacon networks*. The last step in lighting your beacon would be to enlist it in one or more network(s) relevant to your community. Every beacon network would have its protocol for onboarding new beacons; contact the network administrators to find out about these rules. If you are enthusiastic enough, you can trigger the organization of a beacon network with your partners or members of consortia you are part of and customize it to maximally suit that network’s needs!

Contact members of your research community to enquire about existing beacon networks—or start your own! You may use the ELIXIR Beacon network demonstrator (https://beacon-network-demo.ega-archive.org/) for inspiration.

## Conclusion

With a dozen implementations being established within its first 18 months, the Beacon protocol has proven to be a useful, flexible tool for data discovery. At the deployment stage, however, the many possible combinations of granularity and security settings seem somewhat daunting. The above 12 tips will help the reader when making decisions during the deployment of their beacon, every step of the way. The authors of these tips, all involved in the creation of the Beacon standard, are also more than happy to engage with and support interested parties.
